# Timed restricted feeding cycles drive daily rhythms in female rats maintained in constant light but only partially restore the estrous cycle

**DOI:** 10.3389/fnut.2022.999156

**Published:** 2022-09-20

**Authors:** Natalí N. Guerrero-Vargas, Estefania Espitia-Bautista, Rene Escalona, Haydée Lugo-Martínez, Mariana Gutiérrez-Pérez, Raful Navarro-Espíndola, María Fernanda Setién, Sebastián Boy-Waxman, Elizabeth Angélica Retana-Flores, Berenice Ortega, Ruud M. Buijs, Carolina Escobar

**Affiliations:** ^1^Departamento de Anatomía, Facultad de Medicina, Universidad Nacional Autónoma de México, Mexico City, Mexico; ^2^Centro de Ciencias de la Complejidad, Universidad Nacional Autónoma de México, Mexico City, Mexico; ^3^Departamento de Embriología y Genética, Facultad de Medicina, Universidad Nacional Autónoma de México, Mexico City, Mexico; ^4^Instituto de Fisiología Celular, Universidad Nacional Autónoma de México, Mexico City, Mexico; ^5^Laboratorio de Patología, Centro Médico Naval, Mexico City, Mexico; ^6^Departamento de Fisiología Celular y Biología, Instituto de Investigaciones Biomédicas, Universidad Nacional Autónoma de México, Mexico City, Mexico

**Keywords:** circadian rhythms, artificial light at night (ALAN), estrous cycle, restricted feeding schedules, reproductive function

## Abstract

Light at night is an emergent problem for modern society. Rodents exposed to light at night develop a loss of circadian rhythms, which leads to increased adiposity, altered immune response, and increased growth of tumors. In female rats, constant light (LL) eliminates the estrous cycle leading to a state of persistent estrus. The suprachiasmatic nucleus (SCN) drives circadian rhythms, and it interacts with the neuroendocrine network necessary for reproductive function. Timed restricted feeding (RF) exerts a powerful entraining influence on the circadian system, and it can influence the SCN activity and can restore rhythmicity or accelerate re-entrainment in experimental conditions of shift work or jet lag. The present study explored RF in female rats exposed to LL, with the hypothesis that this cyclic condition can rescue or prevent the loss of daily rhythms and benefit the expression of the estrous cycle. Two different feeding schedules were explored: 1. A 12-h food/12-h fasting schedule applied to arrhythmic rats after 3 weeks in LL, visualized as a rescue strategy (LL + RFR, 3 weeks), or applied simultaneously with the first day of LL as a preventive strategy (LL + RFP, 6 weeks). 2. A 12-h window of food intake with food given in four distributed pulses (every 3 h), applied after 3 weeks in LL, as a rescue strategy (LL + PR, 3 weeks) or applied simultaneously with the first day of LL as a preventive strategy (LL + PP, 6 weeks). Here, we present evidence that scheduled feeding can drive daily rhythms of activity and temperature in rats exposed to LL. However, the protocol of distributed feeding pulses was more efficient to restore the day–night activity and core temperature as well as the c-Fos day–night change in the SCN. Likewise, the distributed feeding partially restored the estrous cycle and the ovary morphology under LL condition. Data here provided indicate that the 12-h feeding/12-h fasting window determines the rest-activity cycle and can benefit directly the circadian and reproductive function. Moreover, this effect is stronger when food is distributed along the 12 h of subjective night.

## Introduction

Artificial light at night (ALAN) (1) has brought enormous benefits to human society by changing the outdoor, home, and work environment (2), which enables people to work, walk safely in the streets, and engage in social and leisure activities during the night. However, exposure to ALAN has caused unexpected emerging problems for human and animal health (3, 4). Clinical observations and experimental studies point out that ALAN is a factor leading to disruption of circadian function, which is associated with health disturbances including gastrointestinal, immune, and menstrual irregularities (5).

Experimental studies have provided consistent evidence of the adverse effects caused by ALAN. The results show that constant bright light (LL) or dim light at night can cause arhythmicity by altering directly the activation in the SCN, observed as reduced expression of c-Fos, loss of clock genes rhythms, and the main neuropeptides, all necessary for transmitting circadian rhythms to the body (1, 6–8). The loss of circadian regulation is observed in activity, core temperature, and hormonal regulation like cortisol, melatonin, prolactin, testosterone, or estrogens. Some hormones exhibit either constant high or constant low levels, associated with LL (9–12). Furthermore, LL promotes tumor growth (13), dyslipidemia, and overweight (14, 15). Importantly, early studies reported that LL induces a condition of persistent estrus, in which follicles continue maturing to a preovulatory stage but the ovulation does not occur, resulting in anovulation, continuous high serum estradiol levels, vaginal cornification, and continuous sexual receptivity (9). Moreover, this persistent estrus leads to a polycystic ovary condition (16).

The estrous cycle in rodents is organized by the interaction of at least two oscillatory systems, one driven by the reproductive axis that includes the cyclic secretion of reproductive hormones, and the other is driven by the participation of regulatory cells in hypothalamic nuclei and the ovaries. The second oscillatory system is the circadian system, orchestrated by the suprachiasmatic nucleus (SCN) (17). The interaction of the SCN with the arcuate nucleus (ARC), the anteroventral medial periventricular nucleus (AVPV), and the ovary is necessary for hormonal regulation of the estrous cycle (18) and provides timing to the hypothalamic–pituitary–gonadal axis for the LH surge for ovulation (9) as well as the timing for the sensitivity in the ovary to hormones (19). The production and release of the gonadotropin-releasing hormone (GnRH) are controlled by kisspeptin-producing neurons in the ARC and in the AVPV. The coordinated timing of these hypothalamic nuclei is required for ovulation (17); thus, disruption of circadian rhythms may affect this fine-tuned oscillatory interaction.

Early studies reported that SCN lesions in female rats resulted in anovulation and persistent estrus evaluated by persistent vaginal cornification (20, 21). In a similar way, LL, which results in arrhythmic behavioral patterns, disrupts the estrous cycle (22). Under LL, persistent estrus occurs, characterized by the maturation of follicles to a preovulatory stage without ovulation, and it is associated with continuous high serum estradiol levels, vaginal cornification, and continuous sexual receptivity (9, 23).

In experimental and clinical studies, diverse strategies have been tested to prevent or revert circadian disruption, and this includes scheduled melatonin administration (24), scheduled dexamethasone administration (25), exercise (26), or scheduled feeding (27). Scheduled feeding has shown to be a strong entraining signal when it is coupled to the activity phase. In experimental studies, restricted food access (RF) coinciding with the active phase accelerated resynchronization in a jet-lag model (27, 28) and prevented circadian desynchrony, depressive-like and anxiety-like behaviors, as well as an exacerbated immune response in an experimental model of shift work (29–31). Moreover, in mice exposed to a high-fat diet, RF prevented deleterious effects on metabolism (32–34). In contrast, scheduled feeding that is in conflict with the light–dark cycle exerts detrimental effects on the circadian function and metabolism (35).

In this study, we hypothesized that in female rats exposed to LL, scheduled feeding based on a 12-h feeding/fasting cycle would impose 24 h daily rhythms and consequently would restore the estrous cycle. We tested scheduled feeding as a re-entrainment (rescue) strategy in arrhythmic rats after 3 weeks in LL, or as a preventive strategy by exposing rats to the 12-h feeding schedule simultaneously to the onset of LL. The present study explored RF in female rats exposed to LL, with the hypothesis that this cyclic condition can restore or prevent the loss of daily rhythms and the estrous cycle. The effect of food given in distributed pulses as a stronger entraining rescue or preventive signal was also investigated.

Daily rhythms of general activity and core temperature were monitored, as well as c-Fos day–night patterns in the SCN, in the ARC, and in the AVPV. To test the direct impact on the estrous cycle, vaginal smears were obtained and, at the end of the study, blood samples and ovaries were extracted for hormonal determinations and histological analysis.

## Materials and methods

### Animals and housing

Female Wistar rats weighing 120–150 g were housed in individual acrylic cages (45 cm × 30 cm × 20 cm) placed on tilt sensors, in soundproof ventilated lockers. Rats were maintained under controlled temperature (22 ± 1°C), and they were given free access to water and regular chow (Rodent Laboratory Chow 5001, Purina, Minnetonka, MN, USA), except in the experimental stage of food restriction. During baseline, rats were kept in a controlled 12:12-h light/dark (LD) cycle, lights on at 08:00 h (ZT0). The committee for ethical evaluation at the Facultad de Medicina UNAM approved experiments (FM/DI/140/2019) according to international guidelines for the ethical use of animals. Procedures were aimed at minimizing the number of animals and their suffering.

### Experimental design

#### Experiment 1

The first experiment was explored in female rats exposed to constant light (LL), the potentiality of a timed feeding schedule of 12-h feeding/12-h fasting on activity and temperature circadian rhythms induced or prevented the loss of regular estrous cycles. All rats were first monitored for 1 week of baseline (BL) in a 12:12 LD cycle. For the restitution protocol, a group of rats (LL + RFR; *N* = 9–10) was made arrhythmic by exposing them for 3 weeks to LL (250 lx at cage level). Arhythmicity was defined as the absence of a circadian peak of general activity in the periodogram (Supplementary Figure 1). This was followed by 3 more weeks in LL combined with a 12-h feeding/12-h fasting restricted feeding schedule, which was expected to restore the daily rhythms (LL + RFR). For convenience, the external LD cycle was used as a reference for setting the 12-h food window starting food access at 07:00 am external time.

For the preventive strategy, rats were exposed to the 12-h feeding/12-h fasting restricted feeding schedule simultaneously to the first day of constant light (LL + RFP; *N* = 9–10) for 6 weeks. Rats were exposed to LL, and simultaneously the 12-h feeding/12-h fasting schedule was imposed for 6 weeks. The 12-h feeding started at the previous ZT12 and ended at the previous ZT0.

Two different groups of rats were used. One group was employed for the activity and temperature monitoring and the end for obtaining ovaries and brains. The second group of rats was used for performing vaginal smears, blood samples, and obtaining ovaries and brains.

#### Experiment 2

In the first experiment, the 12-h feeding/12-h fasting schedule partially induced a general activation in the expected active phase. General activation and food intake were mainly seen in the first half of the activity phase (see Supplementary Figure 2). Therefore, in this second experiment, food was provided in four pulses distributed every 3 h during the 12-h feeding window. The hypothesis was that keeping rats active and feeding for 12 h, due to distributed feeding, would provide a stronger entraining signal for the SCN and the circadian function and this would promote regular estrous cycles.

After 1 week in BL, rats were randomly assigned to one of two conditions. The first group of rats (*N* = 9–10) was made arrhythmic by exposing them for 3 weeks to LL. Arhythmicity was confirmed as in experiment 1. This group of rats continued for 3 more weeks in LL combined with a distributed 12-h timed restricted feeding with food distributed in four feeding pulses (LL + PR). Similar to experiment 1, the external LDL cycle was used as a reference for setting the 12-h window starting food access at 07:00 am external time. Thus, it was termed ZT12, and then, food was provided every 3 h to complete the 12 h feeding phase. Meal events were at ZT12, ZT15, ZT18, and ZT21. The second group of rats (*N* = 9–10) was exposed to LL, and simultaneously on the first day of LL, and food was given in four feeding pulses (LL + PP) for 6 weeks as a preventive strategy. To define the amount of food to be delivered every 3 h, the same rats were monitored during their BL to assess the amount of food consumed for 24 h. This amount was divided into four portions of 3.5 g of regular chow pellets for rats distributed during the 12 h of subjective night. Similar to experiment 1, the 12-h food window started at the previous ZT12 and ended at the previous ZT0.

The effects on circadian function were evaluated in all rats, by monitoring general activity and core temperature during the baseline and the 6 weeks of the experimental condition. At the end of the experimental protocol, the day–night c-Fos activation was determined for the SCN, ARC, and AVPV.

To determine the effects on the reproductive function, the second series of rats (*N* = 6–7) was exposed to the same two experimental conditions. Vaginal smears were obtained for 9 consecutive days, three times along the experiment: during BL, after 3 weeks in LL, and after 3 weeks in the restricted feeding schedule. At the end of the experimental protocols, rats were euthanized, and a blood sample, ovaries, and brains were extracted. As control groups, we added two more conditions: (1) rats maintained in 12:12 LD control conditions (*N* = 7) and (2) rats maintained in constant light (LL; *N* = 7) for 3 weeks. Tissues and blood were incorporated for the analysis.

### Activity recording and analysis

General activity in the home cage was continuously monitored with the tilt sensors placed under the cages, and behavioral events were collected with a digitized system and automatically stored every minute in a PC for further analysis with the program SPAD9 (Data processing system, 1.1.1 version; Omnialva SA. De CV. Mexico City, Mexico) based on MATLAB. Double-plotted actograms were obtained by collecting the sum of activity for 15-min intervals. For each experimental stage, 24h mean activity profiles were obtained using data from the last week in the condition. To evidence the day–night activity patterns for each experimental stage, the % of day and night activity counts in 24 h was estimated. Due to the statistical difference between nocturnal activity in LL, LL + RFR, and LL + RFP suggesting a different distribution of the nocturnal activity, the activity counts of the first half were compared with the second half of the night.

### Monitoring of core temperature

One week before starting BL, rats underwent a brief surgery to receive an intra-abdominal temperature sensor (iButton Sensor-Temperature Logger; Maxim integrated products, USA), programmed to store samples every 30 min as previously described. Briefly, rats were anesthetized with an intramuscular dose of xylazine (Procin 8 mg/kg) and ketamine (Inoketam 40 mg/kg). Under anesthesia, a small incision was performed to the dorsal abdominal cavity, and the temperature sensor, previously sterilized, was introduced in the peritoneal region. Muscles were sutured with absorbable catgut (000), and skin was sutured with surgical suture (000 Atramat, International Farmacéutica, SA. de CV. Mexico). Rats were left for 1 week to recover before starting the BL. For each experimental stage, heatmaps were elaborated with the mean temperature of all subjects from the corresponding group. For each experimental stage, 24 h mean temperature profiles were obtained using data from the last week in the condition. To evidence the day–night temperature patterns for each experimental stage, the mean temperature of the day and night was estimated.

### Preparation and analysis of vaginal smears

For each experimental stage, vaginal smears were obtained for 10 days between 10:00 and 11:00 h, representing ZT2-ZT3 of the rat cycle. Vaginal swabs were collected by careful and gentle pipetting of 60 μl of sterile saline solution (0.9% NaCl), approximately 3–5 mm into the rat vagina. Smears were collected on gelatin-coated glass slides and counterstained with hematoxylin–eosin. The cell types contained within each vaginal smear were determined using a light microscope under 40X magnification. The stage of the estrous cycle was determined by observing the predominant cells in the sample as previously reported (36, 37). Briefly, estrus is characterized by the absence of nucleated cells and the presence of cornified squamous epithelial cells, diestrus is characterized by the predominance of leukocytes cells, and proestrus is indicated by the predominance of nucleated epithelial cells. Rats were scored as having regular estrous cycles if they exhibited 4- to 5-day cycles throughout the monitoring period. Conversely, if rats exhibited continuous days in the same stage or did not follow the order of the progression of estrous states, they were scored as having irregular estrous cycles.

The number of days occupied by each stage was represented as % of the 9 sampled days, and the proportion of days occupied by an estrous phase was compared between groups.

### Brain and ovary extraction

At the end of each experiment, all rats were euthanized at either one of two time points, at ZT2 or ZT14. For rats under LL condition, ZTs were based on the time of food intake, considering ZT12 as the time of food access and ZT0 as the time of food removal. After an overdose with pentobarbital (Pisabental, sodic pentobarbital. Euthanasia dose: 65 mg/kg), rats were perfused with 200 ml saline (0.9%), followed by 200 ml of 4% paraformaldehyde in 0.1 mM phosphate buffer (pH 7.2). Brains and ovaries were extracted and were postfixed in 4% paraformaldehyde. After 48 h, brains were cryoprotected in 30% sucrose solution for 5 days and then cut with a cryostat at –18°C in sections of 40 μm and organized in four series.

### Brain immunohistochemistry

One series from each brain was incubated for the c-Fos protein for 72 h (4°C) with the primary antibody anti-c-Fos made in rabbit (1:3000, Millipore) diluted in phosphate-buffered saline (PBS, 0.1 Mmol pH 7.2), 0.25% nutritive gelatin, and 0.5% triton (PBSGT). Brains were processed according to the avidin-biotin method (ABC kit, Vector) and were reacted with 0.05% diaminobenzidine and nickel sulfate, which produced a brown-blue precipitate. After incubations, tissue was rinsed three times in PBS (10 min each). Sections were mounted on gelatin-coated slides, dehydrated in a series of alcohols, cleared with xylene, and coverslipped with Entellan (Merck, Darmstadt, Germany).

The arcuate nucleus, SCN, and AVPV nuclei were identified in the atlas of Paxinos and Watson (38). Three sections were chosen for each region and were counted bilaterally. Microphotographs were obtained under 20X magnification (Supplementary Figures 3, 5) with an optical microscope (LEICA DM500) and a digital camera (LEICA ICC50 HD). Immunopositive c-Fos neurons were counted with the Image J software setting an automatic color and size threshold.

### Histology for ovarian morphometry

Follicular presence was assessed in paraffin sections using one ovary at random from each animal. Ovaries were fixed overnight in 4% paraformaldehyde in PBS, dehydrated in ethanol, and embedded in paraffin. The tissue was serially sectioned into 5 μm thick sections, every fifth section was collected on glass slides, and a total of 5–7 sections per ovary were obtained and analyzed.

The tissue sections were deparaffined and stained with hematoxylin and eosin for follicle counting. Microphotographs were obtained in a Primo Star upright light microscope, using a 10X magnification and an Axiocam ERc 5s digital camera (Zeiss, Oberkochen, Germany). Follicles were classified according to the previously reported criteria (22). Briefly, primary follicles displayed a single layer of cuboidal granulosa cells, and follicles were classified as secondary when more than one layer of granulosa cells was observed without a visible antrum. Finally, Graafian or tertiary follicles were those with an evident antral cavity with a ring of cumulus cells surrounding the oocyte. Only follicles with a visible nucleus in the oocyte in each section were counted, to avoid counting the same follicle more than once. The number of follicles per ovary was calculated as the sum of each follicle category in every section quantified. In addition, the number of cysts for each ovary was determined. Briefly, cysts were identified as those follicles displaying a large antral space lacking an oocyte, surrounded by a dense thecal cell layer with little to none viable granulosa cells. The absence of an oocyte in every section was necessary to classify a follicle as a cyst. The number of cysts is reported as the mean number of cysts per ovarian section.

#### Hormonal determinations

At the end of each experimental stage, blood samples (300 μl) were collected from tail puncture at ZT2 (9:30 h) in Microvette tubes (SARSTEDT AG & Co.) containing an anticoagulant agent (EDTA). The blood was then centrifuged at 7000 rpm for 7 min, and aliquots of 60 μl of plasma were frozen at –45°C for subsequent analysis. The levels of 17 beta-estradiol and progesterone in the plasma were determined using an enzyme-linked immunosorbent assay kit (IBL International GmbH, Hamburg, Germany). The E2/P ratio was estimated for each experimental stage.

### Statistical analysis

Comparison of activity and temperature profiles was compared with a two-way ANOVA for repeated measures (RM). Day–night values for activity and temperature, 1st half and 2nd half of the night data, c-Fos cell count, and the number of follicle types were compared among groups with a two-way ANOVA. The effects on time spent in estrus, ovary weight and the total number of follicles, and estradiol/progesterone ratio were compared among groups with a one-way ANOVA. All ANOVAs were followed by a Tukey or Sidak multicomparison *post hoc* test with α = 0.05.

## Results

### Experiment 1

#### Activity and temperature daily patterns

**FIGURE 1:**
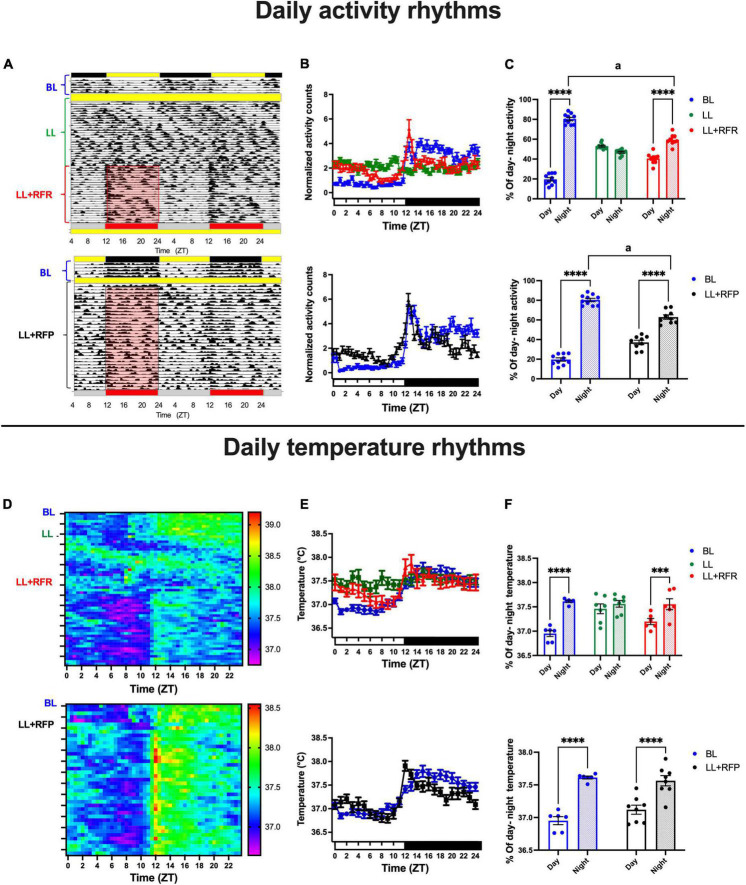
Daily activity and core temperature cycles in rats exposed to a light–dark cycle during the baseline (BL, blue lines and bars), to constant light (LL, green line and green bars), to LL followed by 3 weeks of the 12-h restricted feeding schedule as a rescue strategy (LL + RFR, red), or to LL paired simultaneously to the 12-h restricted feeding schedule as a preventive strategy for 6 weeks (LL + RFP, black). **(A** top) Representative actogram for the BL, LL, and the LL + RFR condition. **(A** bottom) Representative actogram for the BL, LL, and the LL + RFR condition. **(B** top and bottom) Daily activity patterns for the different experimental conditions. Data are expressed as the mean ± SEM; *n* = 9–10/group. **(C** top and bottom) Day–night percentage of activity for the experimental stages; light bars represent day, and dashed bars represent night. **(D** top) Mean heat map for BL, LL, and LL + RFR conditions. **(D** bottom) Mean heat map for the BL and LL + RFP conditions. **(E** top and bottom) Daily temperature patterns for the different experimental conditions; data are expressed as the mean ± SEM; *n* = 6–11/group. **(F** top and bottom) Day–night mean values for core temperature for the experimental stages; light bars represent day, and dashed bars represent night. Asterisk indicates a statistical difference between the day and the night value/group **** = (*P* < 0.0001). The letter a indicates a statistical difference between the nights of each group, a = (*P* < 0001).

During BL, all rats exhibited 24 h daily rhythms adjusted to the LD cycle with low activity counts during the day and high activity counts during the night as can be seen in the representative actogram (Figure 1A top and bottom) and in the mean daily activity profile (Figure 1B top, blue lines). Constant light-induced initially a free-running pattern in all rats that resulted in arhythmicity after 3 weeks of LL exposure (Figures 1A top,B, green line), and by imposing a 12-h feeding schedule (LL + RFR), the general activity acquired a daily activity-rest pattern with increased activation during the 12 h of food access (Figure 1B top, red line). The two-way ANOVA for repeated measures indicated a significant effect for the interaction of time × groups [*F*_(96,1248)_ = 11.25; *P* < 0.0001].

The restricted feeding schedule also prevented an arrhythmic pattern when it was imposed simultaneously with the LL protocol (LL + RFP; Figures 1A bottom, B bottom, black line).

The two-way ANOVA for RM indicated a significant effect on the interaction of time × groups [*F*_(48,768)_ = 7.367; *P* < 0.0001].

The mean proportion (%) of day/night activity corresponding to the 12-h food access/12-h fasting phase indicated that the restricted feeding schedules induced a significant day/night change in general activity (*P* < 0.01; Figure 1C top and bottom). The two-way ANOVA for RM indicated a significant effect for the interaction of time × groups [Figure 1C top: F_(2,26)_ = 129.2; *P* < 0.0001; Figure 1C bottom: F_(1,17)_ = 39.18; *P* < 0.0001]. Importantly, in both conditions, LL + RFR and LL + RFP, the % of nocturnal activity driven by food access remained significantly lower than in the BL (*P* < 0.001).

Similar to that observed in general activity, the core temperature exhibited clear day/night cycles during the BL (Figure 1D). The exposure to LL induced a free-running rhythm for a long period that after 3 weeks resulted in a loss of rhythmicity (Figures 1D top,E top green line). The following 12-h feeding schedule (LL + RFR) induced the recovery of daily temperature cycles with increased levels during the 12 h of food access (Figure 1D top). The two-way ANOVA for RM indicated a significant interaction for time × groups [*F*_(48,360)_ = 7.301; *P* > 0.0001]. When the 12-h feeding schedule was imposed simultaneously with the LL protocol as a preventive strategy (LL + RFP), the loss of rhythmicity of core temperature was prevented (Figures 1D bottom,E bottom, black line). The two-way ANOVA for RM indicated a significant interaction for time × groups [*F*_(24,360)_ = 10.39; *P* > 0.0001].

The 12-h feeding/12-h fasting alternation induced in the mean core temperature a 12-h day/12-h night cycle (*P* < 0.01; Figure 1F top and bottom). The two-way ANOVA for RM indicated a significant effect for the interaction of time × groups [Figure 1C top: F_(2,16)_ = 20.25; *P* < 0.0001; Figure 1C bottom: F_(1,12)_ = 12.59; *P* < 0.0001].

Both the RFR and RFP conditions induced a nocturnal-like activation in rats kept in LL. However, as observed in Figures 1B,C, the intensity of the nocturnal activation was lower and statistically different from the BL condition. A comparison of the 1st half (ZT12–ZT18) and the 2nd half (ZT18–ZT24) of the night activity revealed that for the LL + RFR and LL + RFP groups, a constant nocturnal activation was not completely achieved with the 12-h feeding schedules (Supplementary Figure 2A). For both groups, general activity was significantly lower in the 2nd half of the night as compared to the 1st half of the night (*P* < 0.01). A similar effect was observed when comparing the mean core temperature for the 1st and 2nd half of the night (Supplementary Figure 2B; *P* < 0.01) and food ingestion (Supplementary Figure 2C; *P* < 0.01).

#### Daily-night c-Fos expression in the suprachiasmatic nucleus, arcuate nucleus, and anteroventral medial periventricular nucleus

**FIGURE 2:**
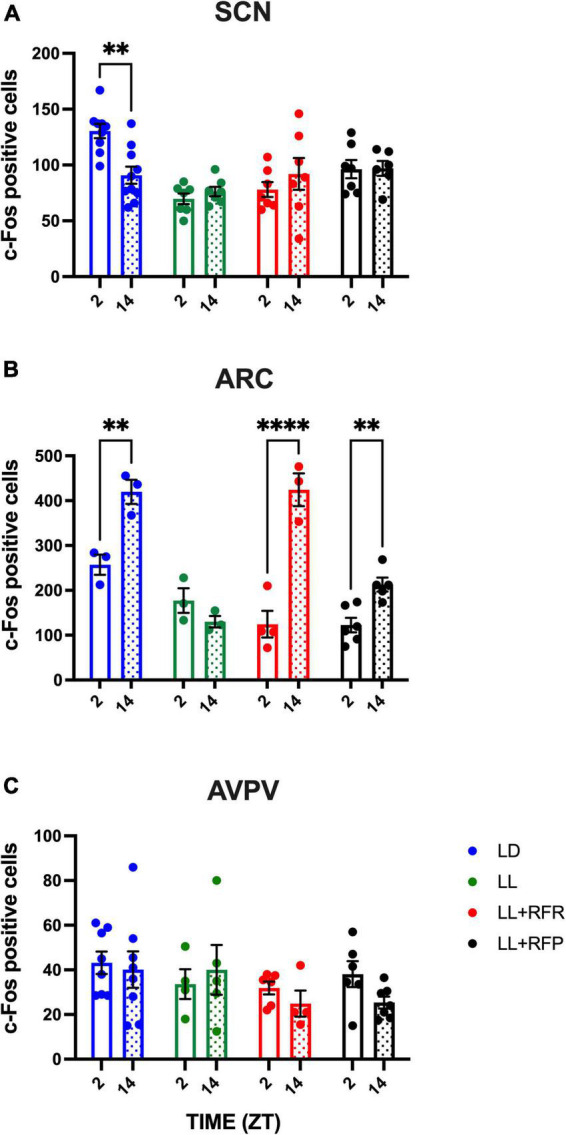
Day–night c-Fos positive cells in **(A)** the suprachiasmatic nucleus (SCN), **(B)** the arcuate nucleus (ARC), and **(C)** the posterior ventral preoptic area (AVPV), in rats exposed to a light–dark cycle during the baseline (BL, blue bars), to constant light (LL, green bars), to LL followed by 3 weeks of the 12-h restricted feeding schedule as a rescue strategy (LL + RFR, red bars), or to 6 weeks LL paired simultaneously to the 12-h restricted feeding schedule as a preventive strategy (LL + RFP, black bars). For each group, empty bars represent day (ZT2) and dashed bars represent night (ZT14). Data are expressed as the mean ± SEM; *n* = 4/time/group. Asterisks indicate a statistical difference between the day and the night values in the specified group; ^**^P < 0.001; ^****^P < 0.0001.

To determine whether the RF can impose a day–night pattern of activity in the disrupted SCN as well as in nuclei involved in feeding/metabolism and in the estrous cycle, c-Fos expressing cells were counted. In the LD group, a clear day–night c-Fos activation was observed in the SCN with high cell counts at ZT2 and lower counts at ZT14 (*P* < 0.01). The constant light condition abolished this day–night variation, and this arrhythmic pattern remained in the LL + RFR and LL + RFP rats despite the cyclic feeding schedules (Figure 2A). The two-way ANOVA indicated a significant effect among groups [*F*_(3,27)_ = 8.726; *P* = 0.0003], no effect in time [*F*_(1,25)_ = 0.6557; *P* = NS], and a significant interaction for groups × time [*F*_(3,25)_ = 5.345; *P* = 0.005].

Likewise, in the ARC, day–night c-Fos activation in the LD group was observed with high c-Fos activation at night as compared to the day (*P* < 0.01) coinciding with the activity and feeding phase. This day–night rhythm was abolished by the LL condition but was reestablished (LL + RFR) and prevented (LL + RFP) by the 12-h feeding schedules (Figure 2B). The two-way ANOVA indicated significant differences among groups [*F*_(3,13)_ = 20.67; *P* < 0.001], in time [*F*_(1, 9)_ = 88.89; *P* < 0.001], and a significant interaction for groups × time [*F*_(3,9)_ = 27.29; *P* < 0.001].

In contrast, in the AVPV, no day–night difference was observed in the LD group nor in the groups exposed to 12-h scheduled feeding (Figure 2C). The two-way ANOVA indicated no significant effects among groups, in time nor for the interaction of factors [*F*_(3,40)_ = 0.6630; *P* = NS]. Representative microphotographs are provided in Supplementary Figure 3.

#### Estrous cycle and ovary morphology

**FIGURE 3:**
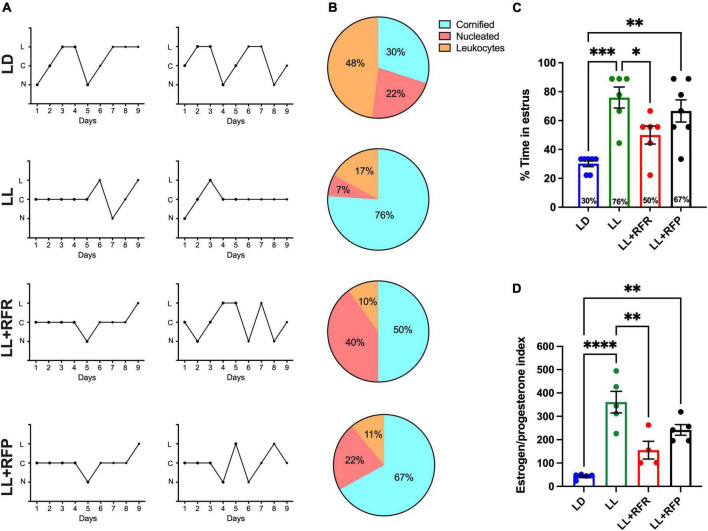
Estrous cycles obtained by 9-day vaginal smears. **(A)** Two representative cycles obtained with the cytology in the vaginal smears of rats exposed to a regular light–dark cycle (LD) from rats exposed to constant light (LL, second top) and rats exposed for 3 weeks to LL followed by 3 weeks of 12-h restricted feeding as a rescue strategy (LL + RFR) and rats exposed to LL simultaneously to RF for 6 weeks as a preventive strategy (LL + RFP). **(B)** Percentage of rats showing predominance for a given cellular type at each day of the estrous stage according to vaginal cytology. **(C)** Proportion of days in which rats displayed estrus. **(D)** Estrogen/progesterone (E_2_/P) ratio. Data are expressed as the mean ± SEM; *n* = 4–5/group. Asterisks indicate *P < 0.05, **P < 0.01, ***P < 0.001; ****P < 0.0001. C, cornified cells; L, leukocytes; N, nucleated cells.

The vaginal smears indicated that 90% of LD rats maintained estrous cycles of 4–5 days (Figure 3A top) from which 30% of the time rats presented the estrous stage (Figures 3B top,C, blue bar). Constant light for 3 weeks resulted in the loss of estrous cycle for all the rats, inducing in 100% of the rats a predominant stage of estrus (*P* < 0.0002). The 12-h feeding schedule following 3 weeks of LL (LL + RFR) partially rescued the estrous cycle (Figure 3A). In the group LL + RFR, all rats remained with irregular cycles (Figure 3B). However, the days in estrus were significantly reduced (Figure 3D) reaching similar values as the LD group and significantly different from the LL group (*P* < 0.04). Imposing feeding schedules simultaneously to the LL as a preventive strategy (LL + RFP) had a similar effect in reducing the days of estrus. However, this was not different from LL (*P* = NS).

The one-way ANOVA indicated a significant difference among groups [*F*_(3,22)_ = 10.23; *P* < 0.0001].

Blood sample analysis indicated a low E_2_/P ratio in LD rats and a significantly increased index in the LL group (Figure 3D). The 12-h restricted feeding reduced this ratio, especially in the LL + RFR females, which reached similar values as the LD group and statistically different values from the LL group (*P* < 0.001). In contrast, the LL + RFP rats remained statistically similar to the LL group and different from the LD (*P* < 0.001). The one-way ANOVA indicated a significant difference among groups [*F*_(3,15)_ = 18.78; *P* < 0.0001].

**FIGURE 4:**
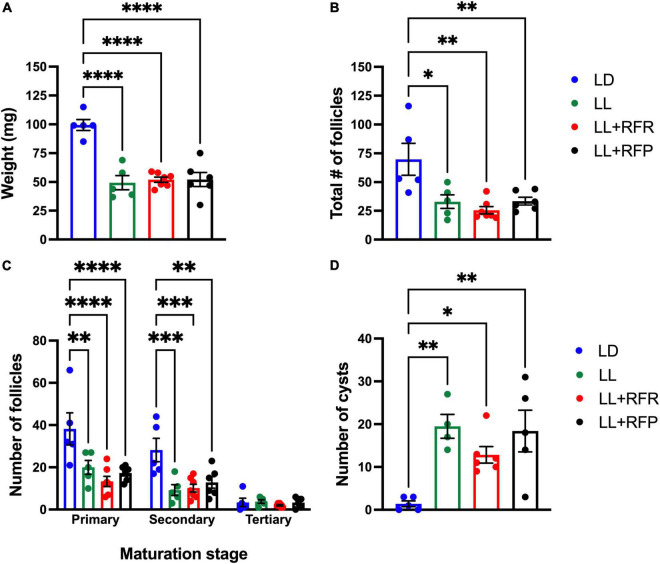
Ovary weight and morphology after LL exposure and the 12-h feeding schedules. **(A)** Ovary weight, **(B)** total number of follicles, **(C)** number of primary, secondary, and tertiary follicles, **(D)** number of cysts. Rats exposed to a regular light–dark cycle (LD, blue), exposed to constant light (LL, green), or exposed for 3 weeks to LL followed by 12-h restricted feeding as a rescue strategy (LL + RFR, red) and rats exposed to LL simultaneously to RF for 6 weeks as a preventive strategy (LL + RFP, black). Data are expressed as the mean ± SEM; *n* = 5–7/group. Asterisks indicate *P < 0.01, ^**^P < 0.001, ^***^P < 0.001 and ^****^P < 0.0001.

After the perfusion, both ovaries were extracted and weighed. Ovaries corresponding to LL rats were significantly smaller than those of the LD group (*P* < 0.001) and no improvement was observed associated with the feeding schedule (Figure 4A). The one-way ANOVA indicated a significant difference among groups [*F*_(3,19)_ = 22.72; *P* < 0.0001], and this was due to a significant difference between groups exposed to LL and their LD control.

**FIGURE 5:**
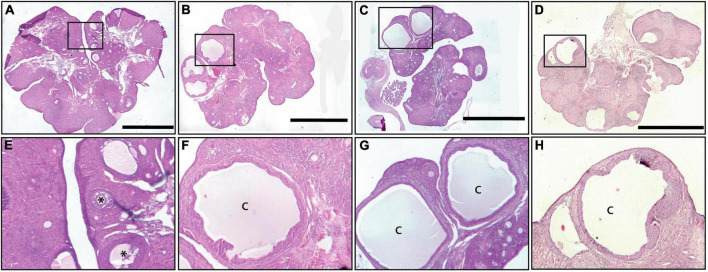
Ovary morphology. An example of a light–dark (LD) control ovary is shown in **(A,E)**, of a constant light (LL) ovary in **(B,F)**, of an ovary from a rat exposed for 3 weeks to LL followed by 12-h restricted feeding as a rescue strategy (LL + RFR) in **(C,G)**, and of an ovary from a rat exposed to LL simultaneously to RF for 6 weeks as a preventive strategy (LL + RFP) in **(D,H)**. Asterisks indicate growing follicles, and “c” indicates cysts. Scale bar = 2 mm.

The histology of the ovaries revealed fewer total follicles in rats exposed to LL as compared to LD rats (*P* < 0.05). The 12-h feeding schedules did not restore (LL + RFR) or prevent (LL + RFP) this effect (Figure 4B). The one-way ANOVA indicated a significant difference among groups [*F*_(3,19)_ = 7.5; *P* < 0.0016]. This difference was mainly due to a reduced number of primary and secondary follicles in all LL groups independent of the feeding schedule. The two-way ANOVA indicated a significant interaction of groups × follicle type [*F*_(6,57)_ = 3.534; *P* = 0.004; Figure 4C]. Remarkably, all groups exposed to LL displayed a significantly increased number of cysts within the ovaries, regardless of the feeding schedule (Figure 4D). Examples of ovarian histology are provided in Figure 5.

### Experiment 2

#### Daily activity and temperature patterns

**FIGURE 6:**
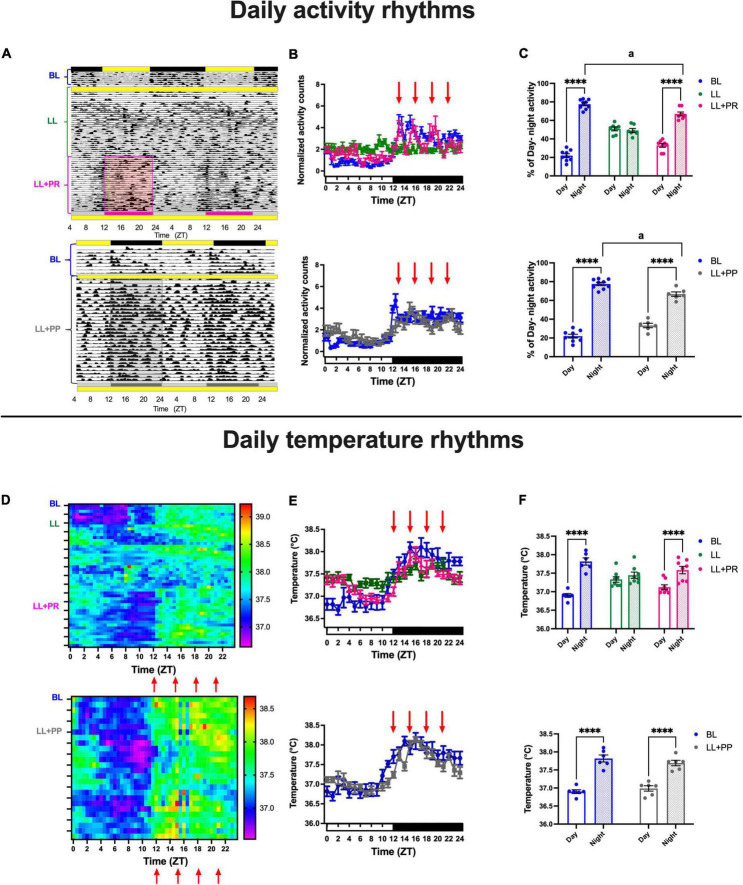
Daily activity and core temperature cycles in rats exposed to a light–dark cycle during the baseline (BL, blue lines, and bars), to constant light (LL, green lines and green bars), to LL followed by 3 weeks of 12-h feeding protocol distributed in 4 pulses feeding events every 3 h as a rescue strategy (LL + PR, pink), or to LL simultaneously to 4 food pulses as a preventive strategy for 6 weeks (LL + PP). **(A** top) Representative actogram from the BL, LL, and the LL + PR condition. **(A** bottom) Representative actogram from the BL and the LL + PP condition. **(B** top and bottom) Mean daily activity profiles for the different experimental conditions: the four feeding pulses indicated by red arrows. Data are expressed as the mean ± SEM; n = 7–9/group. **(C** top and bottom) Day–night percentage of activity for the experimental stages. **(D** top) Mean heat map for the BL, LL, and LL + PR conditions. **(D** bottom) Mean heat map for the BL and LL + PP conditions. **(E** top and bottom) Daily temperature patterns for the different experimental conditions. Data are expressed as the mean ± SEM; *n* = 6–8/group. **(F** top) Day–night mean values for core temperature for the three experimental stages. **(F** bottom) Day–night mean values for core temperature for the two experimental stages of the LL + PP group, light bars represent day, and dashed bars represent night. Red arrows indicate the four events when food was placed in the feeder. Asterisks indicate a statistical difference between the day and the night value/group (****P < 0.0001). For all graphs, light bars represent day (or subjective day) and dashed bars represent night (or subjective night).

As observed in experiment 1, during the BL, all rats exhibited 24 h daily rhythms adjusted to the LD cycle with low activity counts during the day and high activity counts at night (Figures 6A top,B top; blue lines). Similar to experiment 1, rats developed first a free-running pattern that led to arhythmicity after 3 weeks in LL (Figure 6B, green line). Importantly, the activity profile of rats with the distributed food pulses (LL + PR) exhibited increased activation during the four bouts of food access resulting in a continuous activation along the 24 h (Figure 6B top, pink line). The two-way ANOVA for RM indicated a significant interaction time × condition [*F*_(96,768)_ = 5.481; *P* < 0.0001]. Likewise, the distributed feeding schedule induced a 12-h activity pattern when it was imposed simultaneously with the start of the LL protocol (LL + PP), preventing the initial free-running and the following arrhythmic activity pattern (Figures 6A,B bottom, gray line and Supplementary Figure 4A). The two-way ANOVA for RM indicated a significant interaction time × condition [*F*_(48,576)_ = 3.774; *P* < 0.0001].

The mean% of day/night activity corresponding to the 12-h day/12-h night or to the 12-h fasting/12-h food access showed a significant day/night alternation in general activity (*P* < 0.0001) for the BL and the LL + PR (Figure 6C, top). The two-way ANOVA for RM indicated a significant effect on the interaction of time × groups [*F*_(2,21)_ = 50.26; *P* < 0.0001]. Day–night alternation in general activity was also observed in the LL + PP group (Figure 6C bottom). The two-way ANOVA for RM indicated a significant effect on the interaction of time × groups [*F*_(1,13)_ = 14.03; *P* = 0.0024]. Despite the improved 12-h activation achieved with the distributed feeding, in both groups (LL + PR and LL + PP), the % of nocturnal activity remained significantly lower than the BL (*P* < 0.01).

Similar to those observed in general activity, the core temperature exhibited clear day/night cycles during the BL and during the distributed 12-h feeding schedules (Figure 6D). Food distributed in pulses caused a daily temperature cycle with increased levels during the 12 h of food access for both the LL + PR [two-way ANOVA interaction of time × groups *F*_(48,456)_ = 10.06; *P* < 0.0001] and LL + PP groups [Figure 6E: *F*_(24,240)_ = 5.353; *P* < 0.0001].

The mean temperature for the 12-h day/12-h night or for the 12-h feeding/12-h fasting cycles (Figure 6F top and bottom) showed that both experimental groups, LL + PR and LL + PP, reached similar day/night values as the BL group (*P* < 0.0001). The two-way ANOVA indicated a significant interaction for time × groups [Figure 6F top: F_(2,19)_ = 47.73; *P* < 0.0001; Figure 6F bottom: *F*_(1,10)_ = 10.75; *P* < 0.0001].

In contrast to experiment 1, by imposing a distributed access to food, both the LL + PR and LL + PP groups displayed similar nocturnal activation between the 1st half (ZT12–ZT18) and the 2nd half (ZT18–ZT24) of the 12 h of food access (Supplementary Figure 4A) and similar food ingestion (Supplementary Figure 4C).

The two-way ANOVA for RM indicated a significant difference among groups [*F*_(3,22)_ = 30.29; *P* < 0.0001]; however, no significant interaction for time × groups [*F*_(3,22)_ = 1.19; *P* = NS]. A similar effect was observed when comparing the mean core temperature for the 1st and 2nd half of the night for the LL + PR group (Supplementary Figure 4B). However, the LL + PP still exhibited a significant reduction in temperature in the 2nd half of the night as compared with the 1st half (*P* < 0.001). The two-way ANOVA for RM indicated a significant interaction of time × groups [*F*_(3,25)_ = 6.39; *P* = 0.002].

#### Daily- night c-Fos expression in the suprachiasmatic nucleus, arcuate nucleus, and anteroventral medial periventricular nucleus

**FIGURE 7:**
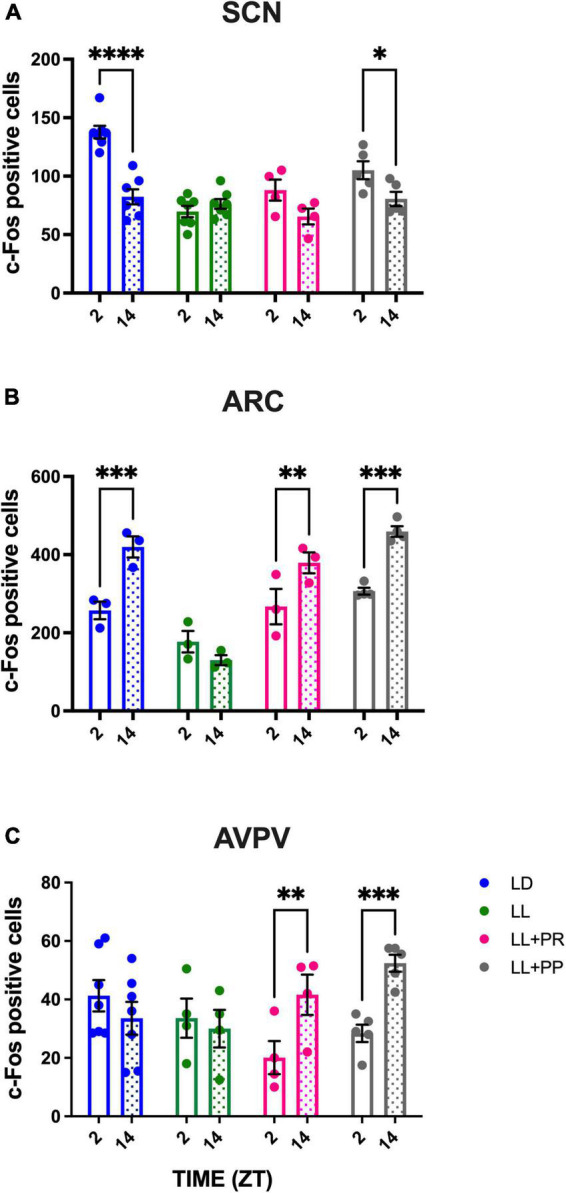
Day–night c-Fos positive cells in **(A)** the suprachiasmatic nucleus (SCN), **(B)** the arcuate nucleus (ARC), and **(C)** the posterior ventral preoptic area (AVPV), in rats exposed to a light–dark cycle during the baseline (BL, blue bars), to constant light (LL, green bars), to LL followed by 3 weeks of 12-h feeding protocol distributed in four pulses feeding events every 3 h as a rescue strategy (LL + PR, pink bars), or to LL simultaneously to four food pulses as a preventive strategy for 6 weeks (LL + PP, gray bars). For each group, empty unfilled bars represent day (ZT2) and dashed bars represent the night (ZT14). Data are expressed as the mean ± SEM; *n* = 3–4/time/group. Asterisks indicate a statistical difference between the day and the night values in the specified group; *P < 0.01, **P < 0.001; *** and **** = *P* < 0.0001.

Under LD conditions, the SCN showed a clear day–night c-Fos activation, with high cell counts for ZT2 and lower counts for ZT14 (*P* < 0.0001). As described for experiment 1, LL abolished this day–night variation. Food distributed in four feeding pulses induced high c-Fos in the subjective day vs. the subjective night (Figure 7A); this was, however, only statistically significant for the LL + PP group (*P* < 0.01). The two-way ANOVA indicated a significant effect among groups [*F*_(3,19)_ = 15.15; *P* < 0.0001], in time [*F*_(1, 19)_ = 034.10; *P* = 0.0001], and a significant interaction for groups × time [*F*_(3,19)_ = 12.23; *P* < 0.0001].

As observed in experiment 1, in the ARC, a day–night c-Fos activation was observed in the LD group with high values during the night as compared to the day (*P* < 0.001), coinciding with the activity, and feeding phase. This day–night rhythm was abolished by the LL condition but was reestablished (LL + PR) and maintained (LL + PP) by the distributed 12-h feeding schedule (Figure 7B). The two-way ANOVA indicated significant difference among groups [*F*_(3,9)_ = 23.16; *P* < 0.0001], in time [*F*_(1,9)_ = 62.29; *P* < 0.0001], and a significant interaction for groups × time [*F*_(3,9)_ = 15.90; *P* = 0.006].

Importantly in the AVPV, the distributed feeding schedules imposed a day–night rhythm with low values during the fasting phase and high values during the feeding (active) phase (Figure 7C). The two-way ANOVA indicated a significant effect in time [*F*_(1,16)_ = 15.24; *P* = 0.0013] and for the interaction of group × time [*F*_(3,16)_ = 15.60; *P* < 0.0001]. Representative microphotographs are provided in Supplementary Figure 5.

#### Estrous cycle and ovarian morphology

**FIGURE 8:**
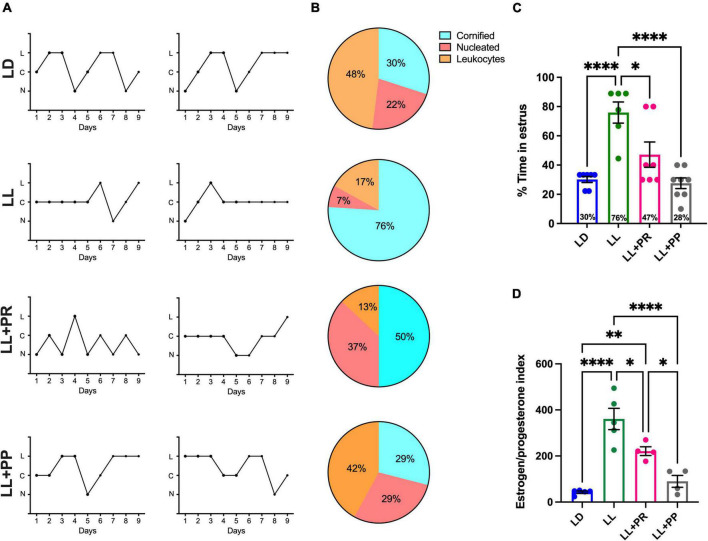
Estrous cycles obtained by 9-day vaginal smears. **(A)** Two representative cycles obtained with the cytology in the vaginal smears of rats exposed to a light–dark cycle during the baseline (BL, blue lines, and bars), to constant light (LL, green lines and green bars), to LL followed by 3 weeks of 12-h feeding protocol distributed in four pulses feeding events every 3 h as a rescue strategy (LL + PR, pink), or to LL simultaneously to four food pulses as a preventive strategy for 6 weeks (LL + PP). **(B)** Percentage of rats showing predominance for a given cellular type at each day of the estrous stage according to vaginal cytology. **(C)** Proportion of days in which rats displayed estrus. **(D)** Estrogen/progesterone (E_2_/P) ratio. Data are expressed as the mean ± SEM; *n* = 4–5/group. Asterisks indicate *P < 0.05, **P < 0.01; ****P < 0.0001. C, cornified cells; L, leukocytes; N, nucleated cells.

Under LD conditions, vaginal smears of 90% of the females indicated estrous cycles of 4–5 days (Figure 8A top) from which 30% of the samples corresponded to the stage of estrus (Figure 8B top,C). As previously shown, LL for 3 weeks resulted in the loss of estrous cycles for all the rats inducing a predominant stage of estrus as compared with the LD group (*P* < 0.0001). The distributed feeding pulses significantly reduced the number of days spent in the estrous stage reaching similar values as the LD group and significantly different from LL (Figure 8C; *P* < 0.01). However, 100% of the rats exposed to LL despite the feeding schedule exhibited an irregular estrous cycle. When comparing the number of days displaying estrous cytology, the one-way ANOVA indicated a significant difference among groups [*F*_(3,24)_ = 13.72; *P* < 0.0001].

Blood sample analysis indicated a low E_2_/P ratio in LD rats and a significantly increased ratio in the LL rats (Figure 8D; *P* < 0.0001). Both conditions of distributed feeding schedules reduced this proportion significantly as compared to LL (LL + PR, *P* = 0.024; LL + PP *P* < 0.0001). However, only the LL + PS group achieved similar values as the LD group. The one-way ANOVA indicated a significant difference among groups [*F*_(3,14)_ = 24.75; *P* < 0.0001].

**FIGURE 9:**
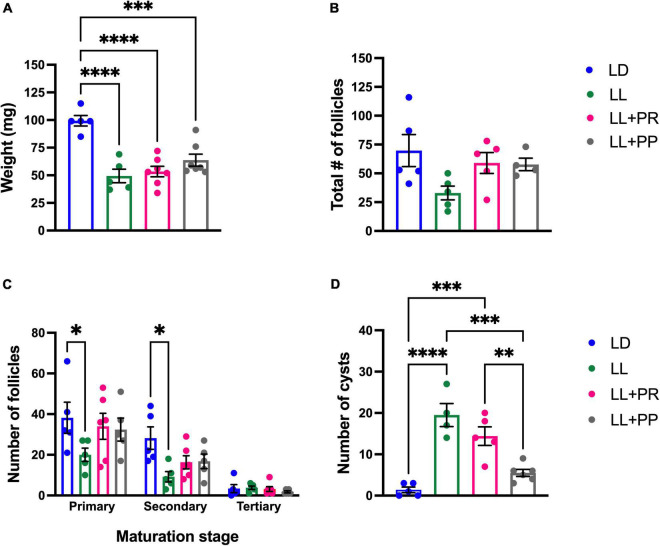
Ovarian weight and morphology after exposition to constant light (LL) and 12-h food distributed in four pulses events every 3 h. **(A)** Ovary weight, **(B)** total number of follicles, **(C)** number of primary, secondary, and tertiary follicles, and **(D)** number of cysts. Data are expressed as the mean ± SEM; *n* = 5–6/group. Asterisks indicate ^****^P < 0.0001.

Ovaries from LL rats were significantly smaller than those of the LD group (*P* < 0.001), and no change was observed associated with the distributed feeding pulses (Figure 9A). The one-way ANOVA indicated a significant difference among groups [*F*_(3, 20)_ = 15.29; *P* < 0.0001].

The histology of ovaries revealed a decreased number of total follicles in rats exposed to LL as compared to LD females. The number of follicles in rats exposed to 12-h distributed feeding pulses exhibited similar values as the LD group (Figure 9B). The one-way ANOVA indicated no significant difference among groups for total follicles [*F*_(3,15)_ = 3.7; *P* = NS]. The effect of the 12-h distributed feeding pulses on the total number of follicles was mainly due to the increased number of primary and secondary follicles (Figure 9C). The two-way ANOVA indicated a significant effect of follicle type [*F*_(2,51)_ = 44.73; *P* < 0.000] and groups [*F*_(3,51)_ = 4.10; *P* = 0.011].

**FIGURE 10:**
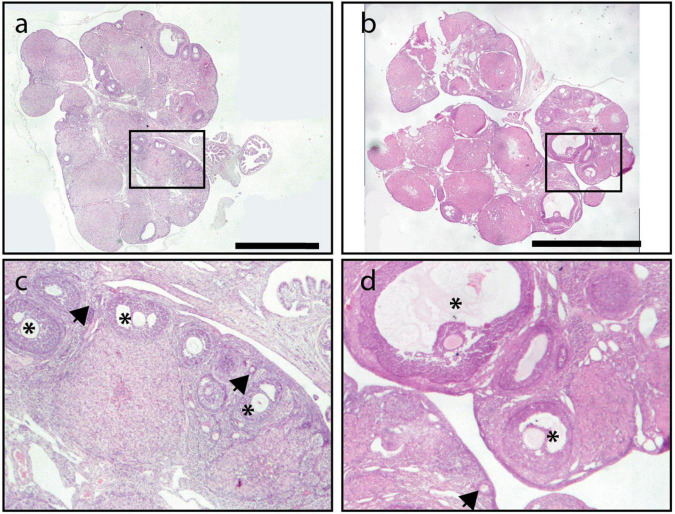
Example of an ovary from a rat exposed to LL followed by 3 weeks of 12-h feeding protocol distributed in four pulses feeding events every 3 h as a rescue strategy (LL + PR) **(a,c)** and an ovary from a rat exposed simultaneously to four food pulses as a preventive strategy for 6 weeks (LL + PP) **(b,d)**. Primary follicles are indicated with arrowheads and growing follicles with asterisks. Scale bar = 2 mm.

Notably, only the preventive strategy (LL + PP) reduced the number of cysts in the ovary as compared to LL rats inducing similar values as the LD group (Figure 9D). The one-way ANOVA indicated a significant difference among groups [*F*_(3,16)_ = 22.39; *P* < 0.0001]. In addition, several corpora lutea were observed in the ovaries of rats in both feeding regimes, suggesting the occurrence of ovulatory cycles. Examples of ovarian morphology can be observed in Figure 10.

## Discussion

Timed restricted feeding is a powerful entraining stimulus for circadian function. Therefore, time-restricted feeding is suggested as a useful strategy to prevent or restore circadian disruption in conditions of experimental shift work or experimental jet lag (27, 29, 39). Also, scheduled feeding ameliorates circadian function under disrupting conditions due to a high-fat diet (40–42), and it improves the response to a glucose tolerance test (43) and is considered an efficient chrononutrition strategy for the treatment of obesity and diabetes (44, 45). Previous studies had evidenced that under constant darkness, feeding schedules can entrain the free-running activity rhythm (46, 47). The present study provides new evidence of the impact that timed restricted feeding can have on restoring disrupted daily rhythms due to ALAN. Here, we show that a cycle of 12-h feeding/12-h fasting can drive 12-h activity-12-h rest cycles of general activity and core temperature. This effect was improved when the access to food was distributed in pulses along the 12 h of scheduled feeding, as seen with the day–night amplitude of general activity and with core temperature. In both experiments, the ARC responded directly to the feeding/fasting cycles as observed with c-Fos activation. However, the AVPV only responded when food access was distributed along the 12 h of the subjective night. Interestingly, a day–night rhythm was observed for the SCN only when food was distributed in pulses along the 12 h of the subjective night as a preventive strategy (LL + PP). Likewise, the LL + PP schedule was more efficient for reducing the days spent in persistent estrus and improving the conditions of the ovary. Our findings confirm that feeding schedules can be an efficient entraining signal and that distributing food intake in pulses along the subjective night comprising a 12-h window that mimics a nocturnal feeding pattern for rodents may impose day–night rhythms on the behavioral, hypothalamic, and reproductive function.

Previous studies have evidenced that disruption of the circadian function in females results in the loss of synchrony for the LH surge and, thus, leads very soon to persistent estrus (23, 48). The estrous cycle requires the synchrony of multiple oscillators, and therefore, the loss of circadian rhythms due to LL would allow oscillators to free-run and drift apart from each other (49, 50). Following this line of thinking, regular 24 h cues might provide the regularity and fine-tuning for the oscillators involved in the reproductive function and therefore revert or prevent the state of continuous estrus under LL. Weber and Adler compared regular schedules with random schedules of vaginal smears in female rats exposed to different constant light intensities and observed that the onset of persistent estrus was delayed but not completely prevented by the daily regular manipulation (49).

Scheduled exercise may be an alternative for restoring disrupted rhythms due to LL. Hughes et al. showed that scheduled voluntary exercise in mice with disrupted SCN activity drives a 24 h rhythm of wheel running and feeding activity (51). Using a model of polycystic ovary syndrome due to prenatal androgen exposure, voluntary wheel running improved the regularity of the estrous cycles, in spite that wheel running was performed *ad libitum* and did not impose a daily scheduled stimulus (52).

Since constant light inhibits melatonin production and melatonin is a relevant internal time signal for the circadian system (53), previous studies have tested the administration of exogenous melatonin as a strategy to restore or prevent the persistent estrus and the anovulatory state associated with LL. In female rats exposed to LL for 1 month, a chronic daily intravenous administration of 100 μl of melatonin induced metestrus and diestrus in 70% of rats, as compared with rats receiving only a single injection (54). Despite driving rats out of the persistent estrous stage, no regular estrous cycles were observed. In our present study, feeding cycles with food distributed in pulses along the subjective night also restored or prevented the persistent estrus in 90% of the female rats, leading rats to metestrus or diestrus, and likewise as observed with melatonin, females exhibited irregular estrous cycles.

Previous studies exploring mechanisms of food entrainment described that the SCN does not shift in response to feeding schedules and is mainly entrained by the light–dark cycle. However, the SCN has the capacity to respond to the metabolic state (fasting or feeding) by modifying its cellular activation as seen with c-Fos (55, 56) and in electrophysiological recordings (57). Also, studies using hypocaloric diets have shown that the SCN can respond to feeding events (58). Exposure to constant light disrupts cellular synchrony in the SCN driving individual cells out of phase. However, in LL, single neurons remain rhythmic (59). The loss of synchrony among VIP neurons may be a condition that facilitates their entrainment to non-photic time signals (60). Thus, the loss of intercellular synchrony in the SCN may have favored that the 12-h feeding/12-h fasting cycle synchronized c-Fos activation in the SCN, which allowed synchrony with other hypothalamic nuclei for the fine-tuning necessary for reproductive function. Importantly, when placing the total food as a single event every day, a constant nocturnal activation was not completely achieved in the LL + RFR and LL + RFP groups. High activation was observed during the first hours of the feeding schedule, and then, a significant decrease occurred. This had important consequences on the response of the SCN and the estrous cycle. In contrast, distributing food in pulses along the 12-h feeding window resulted in a stronger entraining signal for the SCN and the circadian function, and this prevented deleterious effects on the estrous cycle. In this second experiment, the distributed schedule required continuous alertness from the rats, and this factor may have added up to the effect of the feeding schedule as a time signal.

The ovary is tightly driven by the circadian system, and it exhibits molecular clock mechanisms (61, 62); in this regard, the proposed feeding schedules might help to maintain an internal organization of the ovary clock under constant light conditions. In Siberian hamsters (*Phodopus sungorus*), changes in light cycles induced regression of ovarian and uterine size. In mice, 2 weeks of LL caused a reduction of ovarian follicle number accompanied by increased apoptosis of ovarian cells, increased levels of LH, FSH, and E2, and decreased progesterone levels (63). This is in accordance with our observations, and we report a persistent estrous stage, a significant reduction in ovarian size in addition to impaired follicular development, and an increased E_2_/P ratio. Importantly, this condition was reverted with the 12-h feeding cycles, suggesting that in the disruptive circadian function, timed restricted feeding can prevent adverse effects on the ovary.

Interestingly, constant light induces the three major hallmarks of the polycystic ovarian syndrome (hyperandrogenism, polycystic ovarian morphology, and oligoovulation) (64). In our experiments, we observed the development of cysts in LL rats. Remarkably, the scheduled feeding regimes partially reverted these alterations in ovarian morphology. Polycystic ovarian syndrome (PCOS) is one of the most common endocrine disorders in women; despite that, its etiology is poorly understood. In addition, the treatment options for PCOS are not completely efficacious and often cause severe side effects. One emerging factor associated with the development of PCOS is the ALAN, which is affecting a growing proportion of the population. Further studies should explore better the beneficial effects of scheduled feeding regimes as an alternative intervention in the treatment of PCOS.

We provide evidence of the adverse effects of ALAN on reproductive function at the level of the hypothalamus, hormonal ratio, and ovary morphology. We demonstrated that 12-h feeding cycles can drive rest-activity cycles and that they can improve reproductive function. Moreover, forcing rats to eat along the subjective night proved to be more efficient than placing the food once/day in the feeder. Food distributed in pulses resembles better the feeding activity that rats perform during the night. Further studies are necessary to prove that this effect can persist if the 12-h feeding cycles are interrupted or whether the feeding cycle function as a daily hour-clock. Also, in LL conditions, the beneficial effects of the 12-h feeding cycles need to be tested for other systems, like metabolic and inflammatory perturbations described by previous studies (65).

## Data availability statement

The raw data supporting the conclusions of this article will be made available by the authors, without undue reservation.

## Ethics statement

The animal study was reviewed and approved by the Committee for ethical evaluation at the Facultad de Medicina UNAM (FM/DI/140/2019).

## Author contributions

CE: study conception and design and funding acquisition. NG-V, EE-B, RE, HL-M, MG-P, RN-E, MS, SB-W, ER-F, and BO: acquisition of data. NG-V and CE: statistical analysis and drafting of the manuscript. NG-V, EE-B, RE, and CE: analysis and interpretation of data. RB: critical revision. All authors contributed to the article and approved the final version of the article, including the authorship list.
