# Supplementary material for: Timed restricted feeding cycles drive daily rhythms in female rats maintained in constant light but only partially restore the estrous cycle
Source: Front Nutr. 2022 Sep 20;9:999156. doi: 10.3389/fnut.2022.999156 (PMC9531653; doi:10.3389/fnut.2022.999156)
Supplement: Supplementary file 1 [file Data_Sheet_1.pdf]

SUPPLEMENTARY MATERIAL

SUPPLEMENTARY FIGURE 1

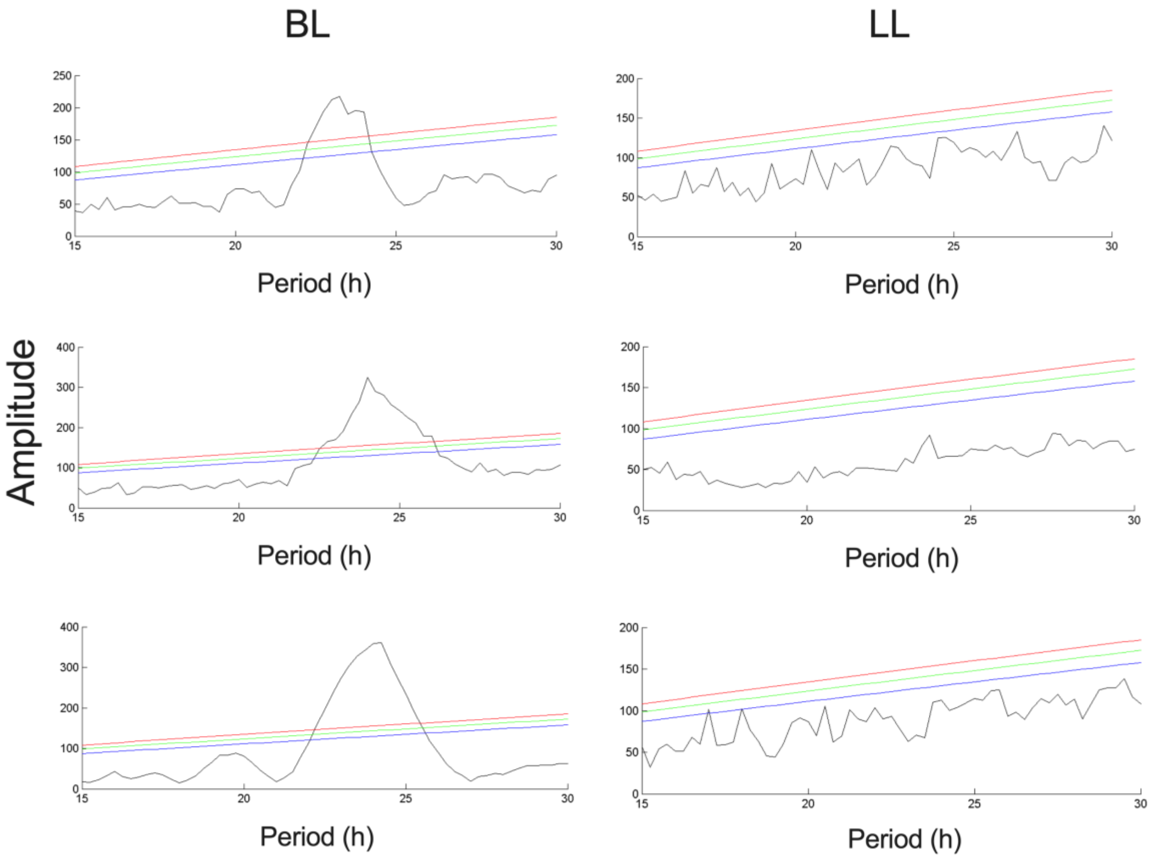

Supplementary Figure 1. Representative periodograms obtained from 3 representative rats exposed to a light dark cycle during the base line (BL, left column) and after 3 weeks of constant light (LL, right column).

SUPPLEMENTARY FIGURE 2

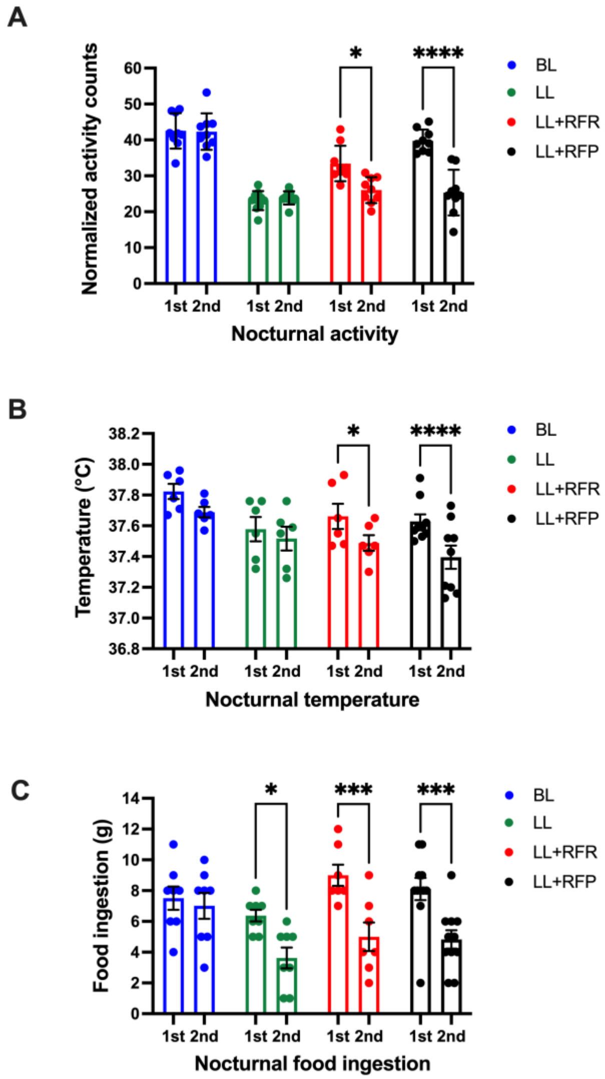

Supplementary Figure 2. Comparison between the first (1st) and the second (2nd) 6 hrs of the nocturnal activity (A) core temperature (B) and of food ingestion (C). In rats exposed to a light dark cycle during the base line (BL), exposed to constant light (LL), or exposed for 3 weeks to LL followed by 12h restricted feeding as a restore strategy (LL+RFR, red) and rats exposed to LL simultaneous to RF for 6 weeks as a preventive strategy (LL+RFP, black). For A data are expressed as the mean ±SEM; n= 9-10/group, for B n=6, for C n= 6-8/group. Asterisks indicate *P=0.01; ***P<0.001; ****P<0.0001.

SUPPLEMENTARY FIGURE 3

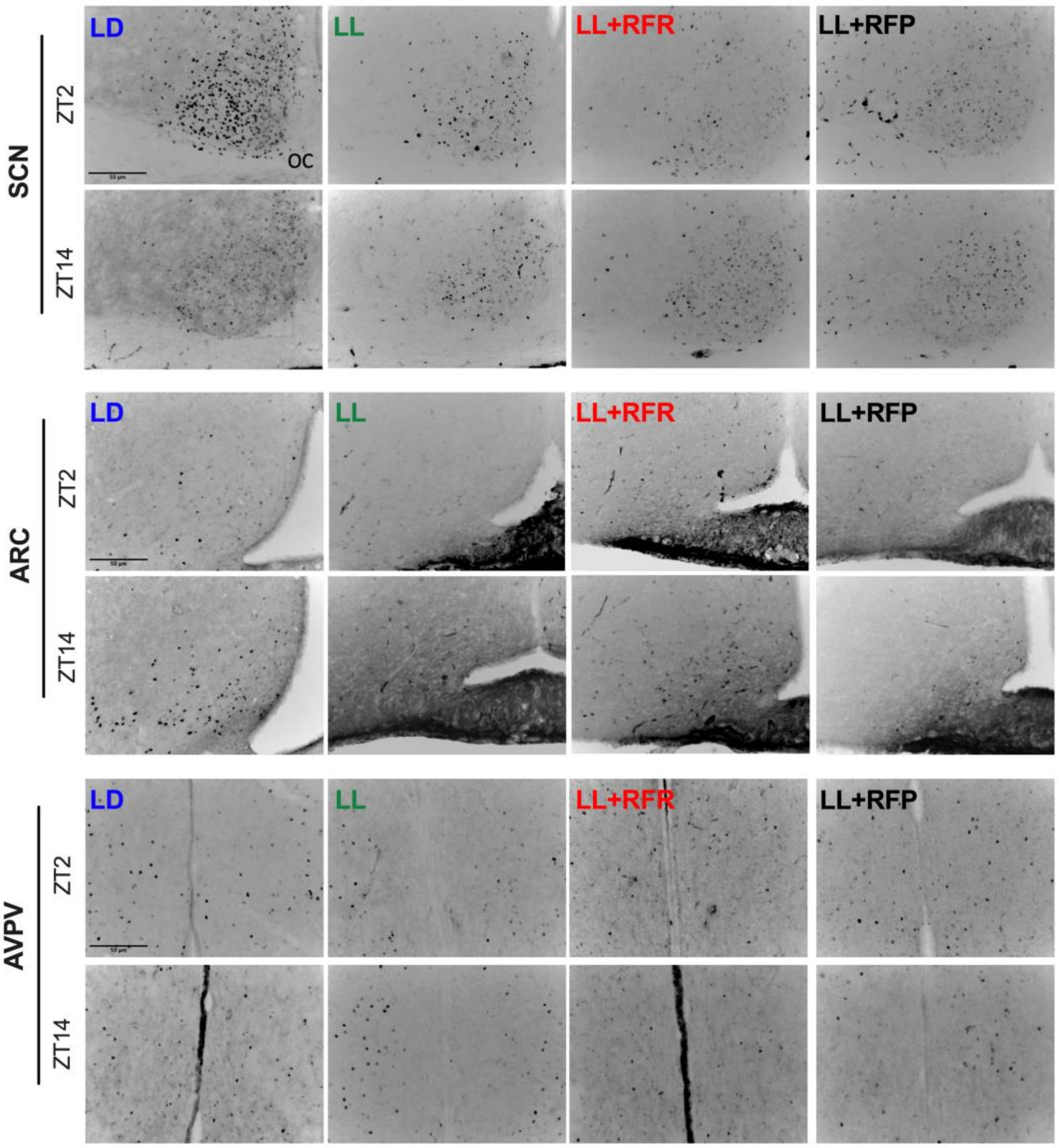

Supplementary Figure 3. Representative microphotographs of c-Fos in the suprachiasmatic nucleus (SCN), the arcuate nucleus (ARC) and the posterior ventral preoptic area (AVPV), in two time points, two hours after the light onset (ZT2) and two hours after lights off (ZT14) in rats exposed to a light dark cycle (LD), exposed to constant light (LL), or exposed for 3 weeks to LL followed by 12h restricted feeding as a restore strategy (LL+RFR) and rats exposed to LL simultaneous to RF for 6 weeks as a preventive strategy (LL+RFP). Scale bar = 50 um.

SUPPLEMENTARY FIGURE 4

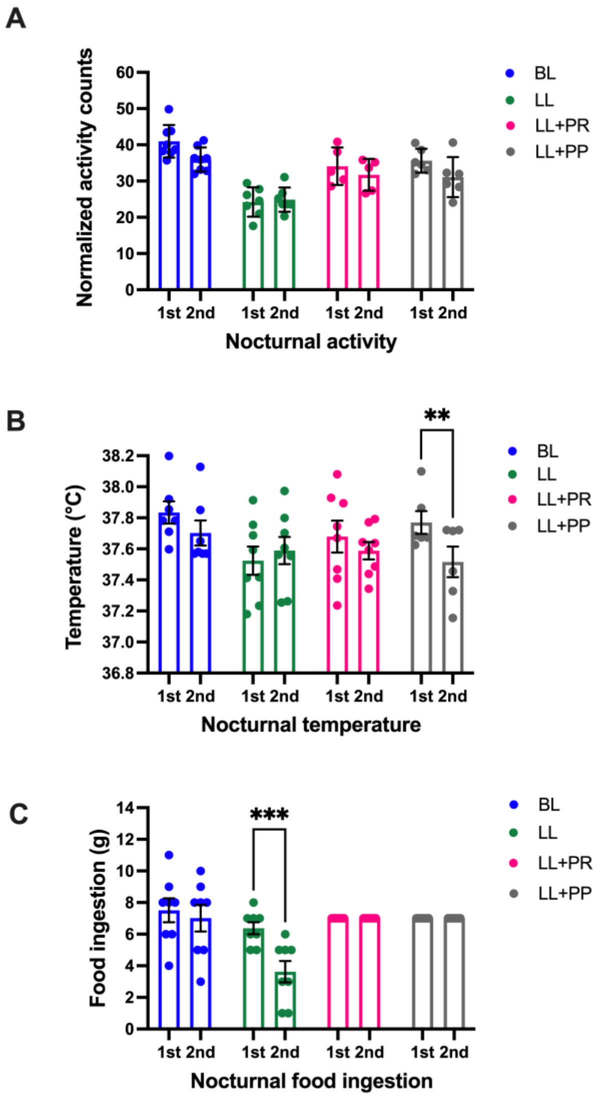

Supplementary Figure 4. Comparison between the first (1st) and the second (2nd) 6h of the nocturnal activity (A) core temperature (B) and of food ingestion (C). In rats during the base line (BL), exposed to constant light (LL), to LL followed by 3 weeks of 12h feeding protocol distributed in 4 pulses feeding events every 3 hours as a rescue strategy (LL+PR) or to LL simultaneous to 4 food pulses as a preventive strategy for 6 weeks (LL+ PP). For A data are expressed as the mean ±SEM; n= 9-10/group, for B n=6, for C n=6-8/group. Asterisks indicate **P<0.01; ***P<0.001.

SUPPLEMENTARY FIGURE 5

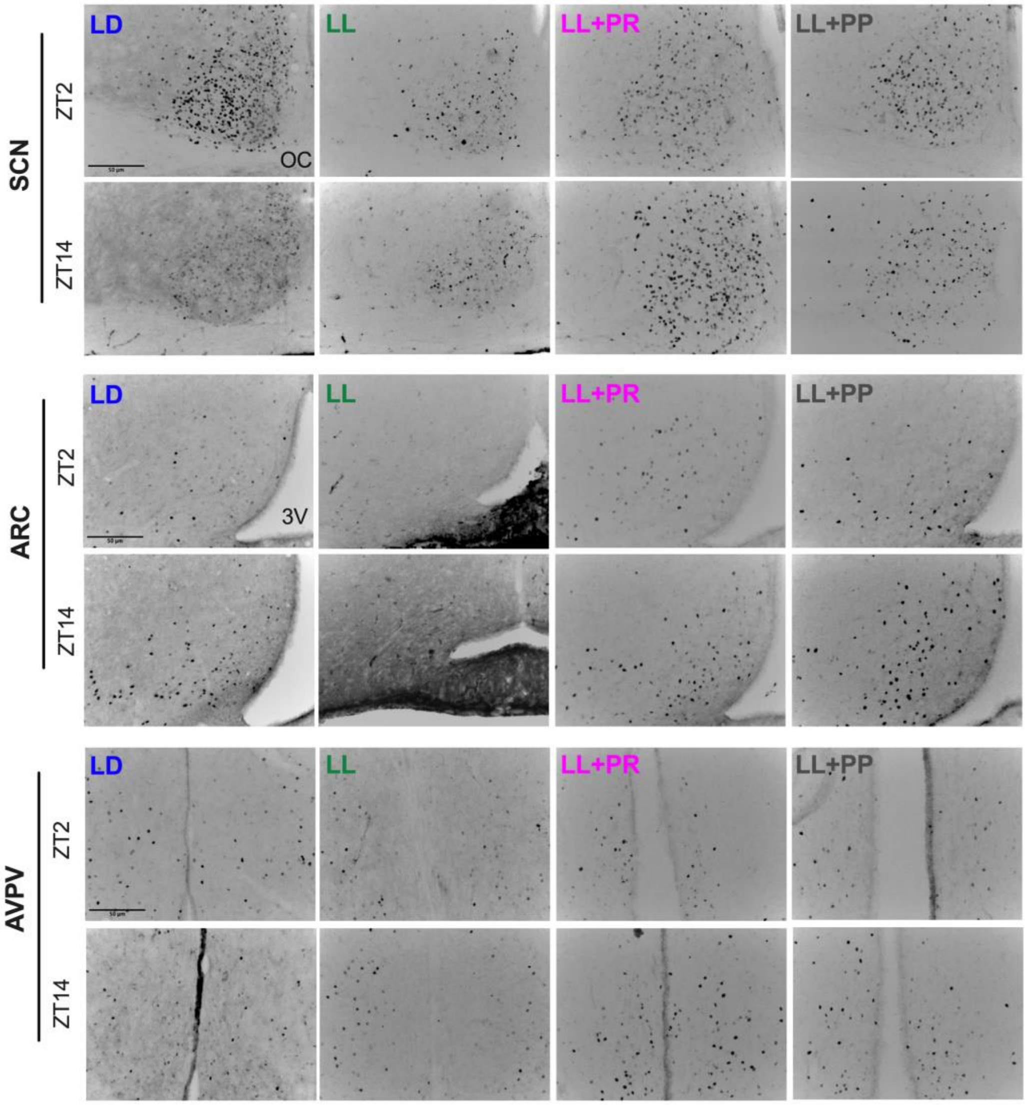

Supplementary Figure 5. Representative microphotographs of c-Fos in the suprachiasmatic nucleus (SCN), the arcuate nucleus (ARC) and the posterior ventral preoptic area (AVPV), in two time points, two hours after the light onset (ZT2) and two hours after lights off (ZT14) in rats exposed to a light dark cycle (LD), to constant light (LL), to LL followed by 3 weeks of 12h feeding protocol distributed in 4 pulses feeding events every 3 hours as a rescue strategy (LL+PR) or to LL simultaneous to 4 food pulses as a preventive strategy for 6 weeks (LL+PP). Scale bar = 50 um.
